# A Predictive Model of Risk Factors for Conversion From Major Depressive Disorder to Bipolar Disorder Based on Clinical Characteristics and Circadian Rhythm Gene Polymorphisms

**DOI:** 10.3389/fpsyt.2022.843400

**Published:** 2022-07-11

**Authors:** Zhi Xu, Lei Chen, Yunyun Hu, Tian Shen, Zimu Chen, Tingting Tan, Chenjie Gao, Suzhen Chen, Wenji Chen, Bingwei Chen, Yonggui Yuan, Zhijun Zhang

**Affiliations:** ^1^Department of Psychosomatics and Psychiatry, School of Medicine, Zhongda Hospital, Southeast University, Nanjing, China; ^2^Key Laboratory of Developmental Genes and Human Disease, Ministry of Education, Institute of Life Sciences, Southeast University, Nanjing, China; ^3^Department of General Practice, School of Medicine, Zhongda Hospital, Southeast University, Nanjing, China; ^4^Department of Epidemiology and Biostatistics, School of Public Health, Southeast University, Nanjing, China; ^5^Department of Neurology, School of Medicine, Zhongda Hospital, Southeast University, Nanjing, China

**Keywords:** bipolar disorder, conversion, circadian rhythm, machine learning, predictive model

## Abstract

**Background:**

Bipolar disorder (BD) is easy to be misdiagnosed as major depressive disorder (MDD), which may contribute to a delay in treatment and affect prognosis. Circadian rhythm dysfunction is significantly associated with conversion from MDD to BD. So far, there has been no study that has revealed a relationship between circadian rhythm gene polymorphism and MDD-to-BD conversion. Furthermore, the prediction of MDD-to-BD conversion has not been made by integrating multidimensional data. The study combined clinical and genetic factors to establish a predictive model through machine learning (ML) for MDD-to-BD conversion.

**Method:**

By following up for 5 years, 70 patients with MDD and 68 patients with BD were included in this study at last. Single nucleotide polymorphisms (SNPs) of the circadian rhythm genes were selected for detection. The R software was used to operate feature screening and establish a predictive model. The predictive model was established by logistic regression, which was performed by four evaluation methods.

**Results:**

It was found that age of onset was a risk factor for MDD-to-BD conversion. The younger the age of onset, the higher the risk of BD. Furthermore, suicide attempts and the number of hospitalizations were associated with MDD-to-BD conversion. Eleven circadian rhythm gene polymorphisms were associated with MDD-to-BD conversion by feature screening. These factors were used to establish two models, and 4 evaluation methods proved that the model with clinical characteristics and SNPs had the better predictive ability.

**Conclusion:**

The risk factors for MDD-to-BD conversion have been found, and a predictive model has been established, with a specific guiding significance for clinical diagnosis.

## Introduction

Bipolar disorder (BD), including depressive and manic phases, is a common mental disorder with high morbidity, disability, and suicide risk, which seriously impairs social functioning (1–3). The probability of depressive episodes in BD patients is 3 times more likely than that of manic episodes (3), and the symptoms of the first depressive episodes of BD are not significantly different from those of major depressive disorder (MDD) (4), so misdiagnosis as MDD is common at the early stage of BD (5). According to Undurraga et al., the interval between the delay to diagnosis of BD is about 6–8 years (6). BD patients who are misdiagnosed with MDD receive inappropriate therapy, which may increase the risk of suicide and have a poorer prognosis (7, 8). There are several reasons for the delay in diagnosis of BD. One is the diversity of the disease itself and another is that the symptoms are insufficient to identify manic episodes (9). Moreover, the lack of identifying risk factors for conversion from MDD to BD is also a reason for the delay (9). Therefore, it is important to provide an early warning and detection of the disease.

Some reports have identified risk factors that are related to conversion to BD, most of which are clinical risk factors, such as the age of onset of the MDD (10), family history of affective disorders (10, 11), suicide attempts (12), psychotic symptoms (13), the number of hospitalizations (14), etc. In addition, dysfunction of 24-h circadian rhythms is also a risk factor (7). Many studies have shown that patients with BD have circadian rhythm dysfunction. Patients with BD have decreased sleep requirements during manic episodes, and the sleep-wake phase delay is common (15). Yoshikazu et al. have shown that patients with BD have a higher rate of circadian rhythm dysfunction symptoms than patients with MDD (7). Circadian rhythm dysfunction is closely related to BD (16), and several studies reveal that circadian rhythm dysfunction is associated with the pathophysiology of BD (17, 18). In mammals, the hypothalamus's suprachiasmatic nuclei (SCN) act as a master circadian pacemaker (19, 20). Regulation at the molecular level mainly relies on transcriptional-translational feedback loops of circadian rhythm genes (21, 22). The main loops include CLOCK, ARNTL (including BMAL1, MOP3, etc.), PER (PER1, PER2, PER3), and CRY (CRY1, CRY2) genes (15). Polymorphisms of these genes may be associated with BD. It has been shown that the circadian rhythm gene pathway is involved in the development of BD (23). Therefore, many genetic studies have attempted to determine the possible association between circadian rhythm gene variation and BD (15). It has been found that mice with CLOCK gene knockout or mutations in the CLOCK gene show manic-like behavior (21). Park et al. have found that the polymorphisms of the circadian rhythm genes TIMELESS and CSNK1E are significantly related to BD (15). Banach et al. have found that the CC genotype of rs11600996 of the gene ARNTL and the TT genotype of rs228642 of the gene PER3 is closely associated with patients with BD and alcohol dependence (23). The previous case-control design showed that the genotype frequencies of rs228642 of PER3 were associated with BD (24). Daytime dysfunction in the Pittsburgh Sleep Quality Index (PSQI) was significantly associated with rs228642 in PER3 in patients with MDD and BD (25), suggesting that rs228642 in PER3 polymorphisms influences sleep quality, especially in patients with MDD and BD. However, no studies have investigated the relationship between circadian rhythm gene polymorphism and MDD-to-BD conversion.

Machine learning (ML) studies complex distributions and determines possible connections based on complex and conditional relationships between variables. In addition, it verifies the dependability of the test results through repeated cross-validation (26). In recent years, the use of ML in the psychiatric field has been increasing, and predictive models have the potential to improve diagnosis and treatment (27). Currently, many studies have established predictive models to help identify and diagnose diseases. A risk stratification model for conversion from MDD to BD was established using a classification and regression tree (CART), including clinical factors such as outpatient follow-up times (28). However, the clinical factors included are not comprehensive. It is worth pointing out that the stratification model utilizes a single site, and thus needs to be verified (28). Another study with ML methods used neuroimaging data to distinguish unipolar depression and bipolar depression, and the classification model established has certain guiding significance for the clinical identification of BD (29, 30). On the other hand, some studies with ML make use of inflammation markers and intestinal microbes to help identify and diagnose BD (31, 32). However, no studies have focused on combining clinical and genetic factors to establish a predictive model for the risk factors for MDD-to-BD conversion.

This study aimed to find clinical risk factors for MDD-to-BD conversion. Based on that, we tried to establish a predictive model through ML by combining clinical and genetic factors. We hypothesized that by combining clinical characteristics with circadian rhythm gene polymorphisms, the predictive model could aid in the accurate clinical identification of BD. In this study, we compared the clinical characteristics of patients from MDD and BD groups to identify clinical risk factors. Then, we selected clinical and genetic features by three methods. Finally, we established a predictive model and evaluated its predictive ability.

## Methods

### Subjects

This study was carried out among inpatients at Zhongda Hospital. We recruited 500 patients with an initial diagnosis of MDD, and their clinical variables were collected to establish a database. The inclusion criteria for MDD were shown as follows. All recruited subjects were between 15 and 65 years old, having presented with depressive symptoms and received an initial diagnosis of MDD from two senior psychiatrists according to the Diagnostic and Statistical Manual of the American Psychiatric Association (DSM-IV). All were initially inpatients and scored 18 or over on the 17-item Hamilton Depression Rating Scale (HDRS-17) (33).

A cohort of 299 of the initially recruited subjects was followed up successfully after 5 years and the diagnosis was determined independently by two senior psychiatrists. Of these 299 subjects, those with a score of more than 6 on the Young Mania Rating Scale (YMRS) (34) met the inclusion criterion for a diagnostic conversion to BD, with the remainder confirmed in their diagnosis of MDD.

Exclusion criteria for MDD included documented history of diagnoses on Axis 1 (including bipolar disorder, schizophrenia, schizoaffective disorder, generalized anxiety disorder, panic disorder or obsessive-compulsive disorder, intellectual disability, personality disorder, substance misuse, alcohol, and drug dependence history) of DSM-IV, pregnancy, lactation, abnormalities of blood measures, heart, liver and kidney function after examination, or a history of brain organic diseases and endocrine diseases. The exclusion criteria of the BD group were the same as those of the MDD group, except for the diagnosis of BD. This study was approved by the Ethical Committee of Zhongda Hospital (2021ZDSYLL062-P01), in accordance with the Declaration of Helsinki. The sample size was calculated by G Power. A two-sample *t*-test was carried out to check for differences between two independent means (two groups). The input parameters included: tails two; parent distribution normal; effect size d 0.5; α err prob 0.05; power (1– β err prob) 0.08; allocation ratio N2/N1 1. Each group should have more than 64 patients.

A previous similar cohort study showed the rate of conversion from MDD to BD in 3 years as 12.4% (35). Assuming an expected rate of conversion of 10–15% in the current study, a cohort of 299 subjects would not yield enough subjects converting to BD to meet the sample size required by the power analysis. To address this, from a retrospective review of inpatient medical records, another 34 patients were identified who developed manic symptoms following an initial diagnosis of MDD. These patients also provided signed informed consent and were interviewed independently by two senior psychiatrists to confirm a current diagnosis of BD with a score of more than 6 on the YMRS. The 34 patients also met the inclusion criterion for a diagnostic conversion from MDD to BD.

In the patients with a stable diagnosis of MDD, a subgroup of 70 subjects was selected by random sampling. There was no significant difference in general clinical characteristics between these 70 patients with MDD and the patients who were not selected. The clinical characteristic variables in both MDD and BD groups included gender, the age of the first onset of MDD, family history of affective disorder, suicide attempt, psychotic symptoms, and the number of hospitalizations.

### Single Nucleotide Polymorphisms (SNPs) Selection and Genotyping Methods

According to the circadian rhythm pathway, 6 candidate genes were selected: CLOCK, ARNTL, CSKN1E, PER1, PER2, and PER3 (36). In total, 78 SNPs were finally selected, using dbSNP (Map to Genome build: 36.3) and HapMap database (Public Release #23) through literature review and site function.

Fasting venous blood was collected from all subjects at 8:00 in the morning into an EDTA anticoagulant tube and stored at −80°C until genotyping. The genomic deoxyribonucleic acid (DNA) was extracted from blood cells by that TIANamp Blood DNA Extraction Kit (Tiangen) and divided into pieces for testing.

Genomic DNA was sheared and subjected to Illumina paired-end DNA library preparation. All the SNPs were genotyped using FastTaget. The captured libraries were loaded onto the Illumina HiSeq platforms. Sequencing was performed at Genesky Biotechnologies Inc., Shanghai, China. The obtained data was directly imported into GenomeStudio software for analysis to obtain the genotype data.

### Statistical Analysis

SPSS version 25 was used to analyze the risk factors for the clinical characteristics. The age of first onset and number of hospitalizations, which did not conform to the normal distribution, were performed using a non-parametric test. Using a chi-square test, categorical variables, including gender, family history, suicide attempts, and psychotic symptoms, were tested. *P* < 0.05 was considered statistically significant. Haploview 4.1 program was used to test for gene detection rate and minor allele frequency (MAF). The SNPs with a detection rate lower than 90% or MAF lower than 5% were eliminated (Supplementary Table S2). We normalized the original data. Each continuous variable was given values between 0 and 1. The three algorithms included random forest (RF), gradient boosting decision tree (GBDT) and recursive feature elimination (RFE) were used for feature selection. The factors included in three machine learning algorithms were gender, the age of the first onset of MDD, family history of affective disorder, suicide attempts, psychotic symptoms, the number of hospitalizations, and 59 SNPs in the Supplementary Data. The results from these three algorithms were overlapped to obtain the important features. Based on the final feature selection results, the R Programming Language (package “rms”) was used to establish the risk prediction model. The predictive model was established by logistic regression, and the model evaluation was performed by the area under curve (AUC), calibration, net reclassification improvement (NRI), and decision curve analysis (DCA). A nomogram was finally used to visualize the model that could calculate the disease's risk probabilities.

## Results

**Figure 1:**
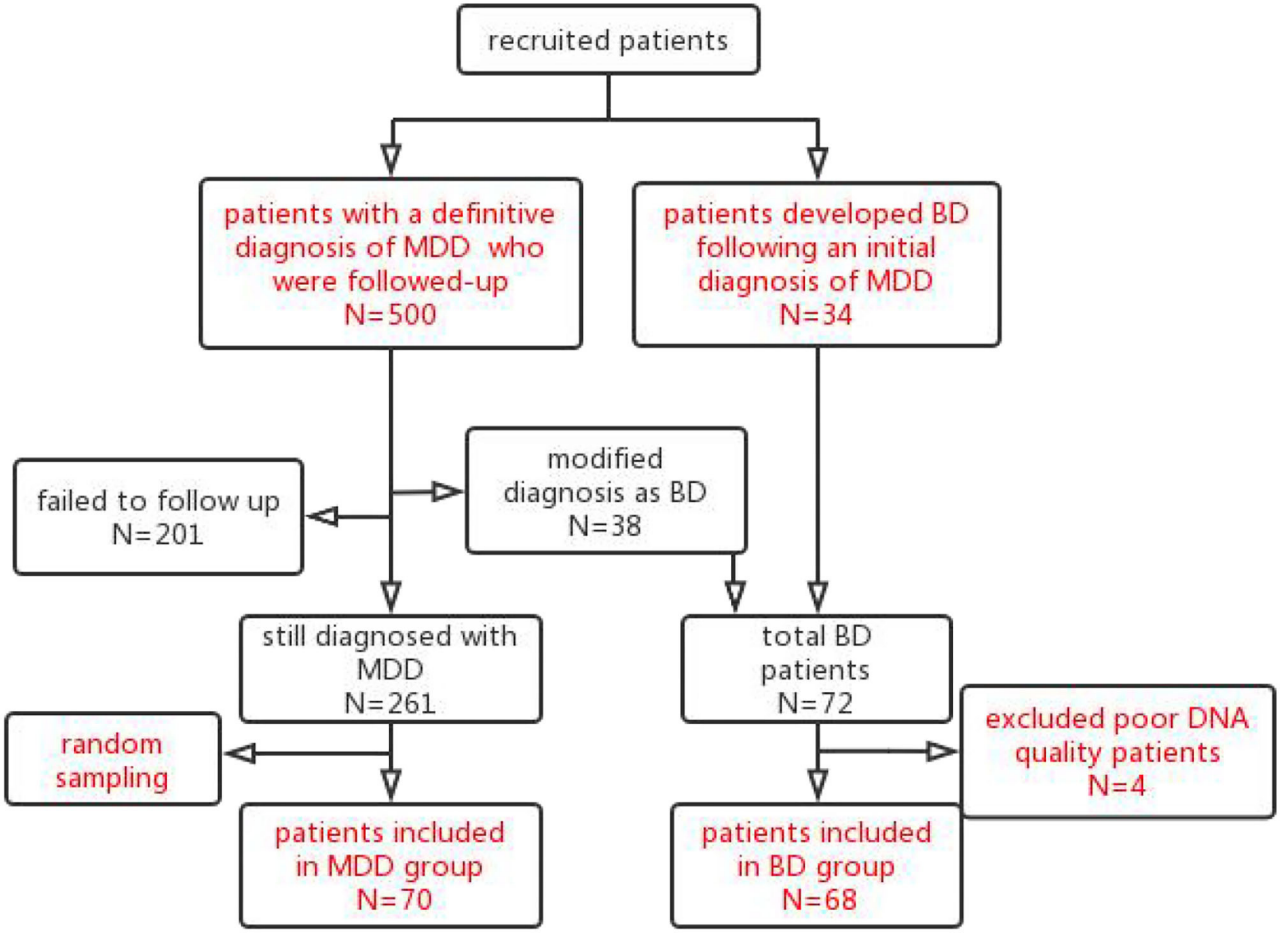
Patients flow chart throughout the trial. The 500 patients with an initial diagnosis of MDD were recruited and followed up after 5 years. In total, 299 subjects were followed up successfully. Of these 299 subjects followed up successfully, 38 subjects met the criteria for a diagnosis of BD and were included in the BD group. Furthermore, from a retrospective review of inpatient medical records, another 34 patients were identified who developed manic symptoms following an initial diagnosis of MDD and were included in the BD group. Of the total 72 BD patients, 4 patients with poor DNA quality were excluded. Finally, 68 patients were included in the BD group. Of the 261 patients with MDD who followed-up successfully with a stable diagnosis of MDD, a subgroup of 70 subjects was selected by random sampling for the MDD group.

Of a total of 299 subjects successfully followed up, 38 were diagnosed with BD, with a conversion rate of 12.7%. Finally, an MDD group of 70 subjects and, after including the second cohort of 34 patients who underwent an MDD to BD diagnosis conversion, a BD group of 72 subjects (Figure 1) underwent further genetic investigation. In terms of clinical factors, the 299 patients successfully followed-up with did not differ from the 201 patients lost to follow-up (Supplementary Table S1). Of the 78 SNPs, 11 had a MAF of 5%, 6 had a detection rate <90%, and 3 had an HWpval of 0.05. One SNP had both a MAF of 5% and a detection rate <90%, leaving 59 SNPs for inclusion (Supplementary Data, Supplementary Table S2). Poor DNA quality prevented genetic analysis for four subjects in the BD group.

### General Information

There were significant differences between the two groups in age at the first onset, suicide attempt, and psychotic symptoms (*P* < 0.05) (Table 1). The logistic regression analysis showed that the age of the first onset was a significant risk factor (Table 2).

**Table 1:** General characteristics of patients with MDD and BD.

	**MDD (*N =* 70)**	**BD (*N =* 68)**	***P*-value**
Gender (male/female)	18/52	28/40	0.054
Age of onset (mean±SD)	46.01 ± 14.372	32.84 ± 15.830	0.000
Family history (yes/no)	57/13	47/21	0.093
Suicide attempt (yes/no)	47/23	33/35	0.027
Psychotic symptoms (yes/no)	3/67	11/57	0.021
The number of hospitalizations (mean ± SD)	1.83 ± 1.383	2.35 ± 2.490	0.170

**Table 2:** Logistic regression of clinical characteristics between patients with MDD and BD.

	***B***	**BE**	**D*f***	***P***	**EXP**	**95%CI**
age of onset	−0.051	0.013	1	0.000	0.950	0.927–0.974
constant	0.100	1.121	1	0.929	1.105	

### Machine Learning

The result of characteristics selected by RFE included gender, age at the first onset, suicide attempt, the number of hospitalizations, rs4146388 in ARNTL, rs1056547, and rs9312661 in CLOCK, rs10462024 and rs2585408 in PER1, rs2304671 and rs10462023 in PER2. The AUC value was 0.852.

A GBDT method was used to select the top ten variables according to the importance of the variables: gender, age of the first onset, suicide attempt, the number of hospitalizations, rs4146388 in ARNTL, rs1056547, rs9312661 and rs3736544 in CLOCK, rs228669 and rs2859387 in PER3. The AUC value was 0.802.

For RF with 5 cross-validation results, according to the smallest Gini index, the top five variables in each classification were chosen. They were gender, age of the first onset, suicide attempt, the number of hospitalizations, rs4146388 in ARNTL, rs2585405 in PER1. The AUC value was 0.616.

### The Predictive Model

**Figure 2:**
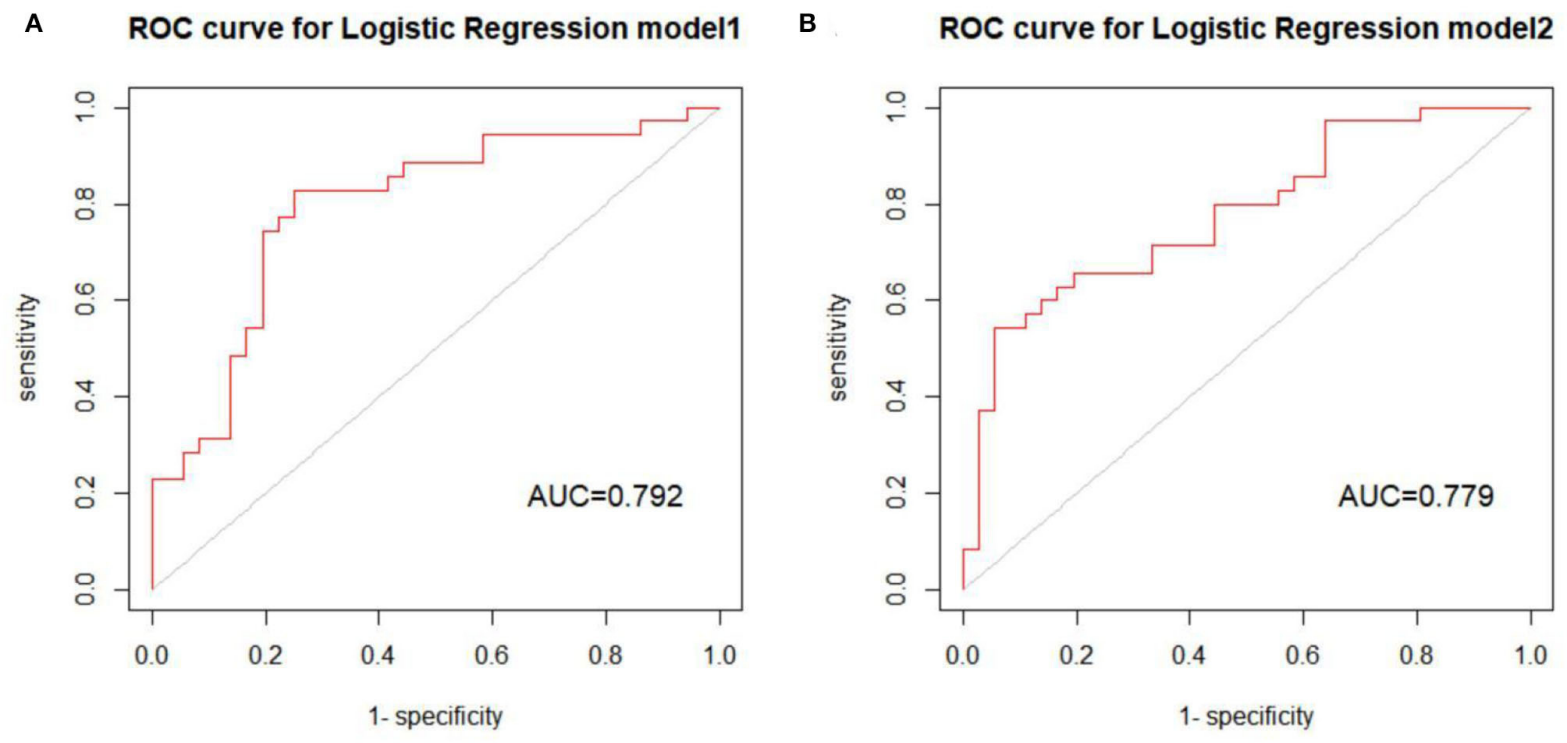
**(A)** The ROC curve and AUC value of model1. **(B)** The ROC curve and AUC value of model2.

**Figure 3:**
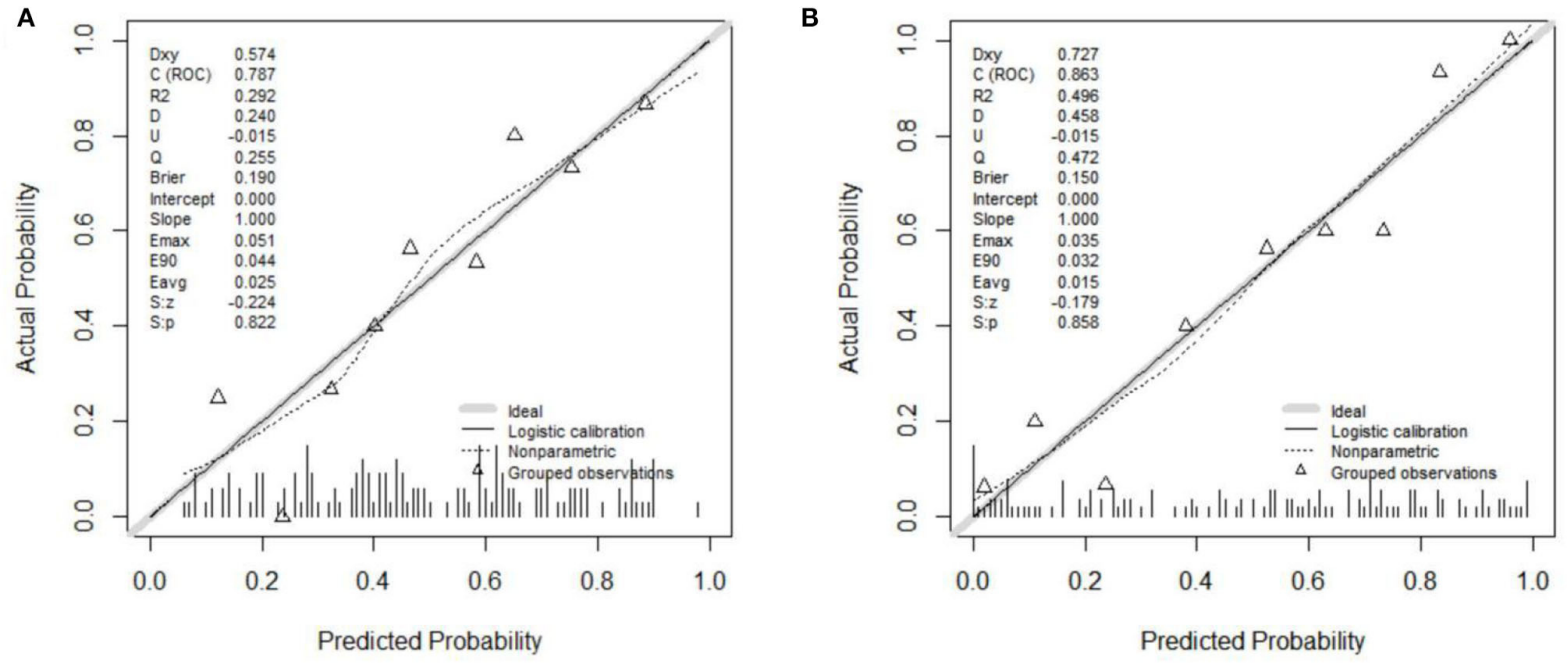
**(A)** The calibration curve of model1. **(B)** The calibration curve of model2.

**Figure 4:**
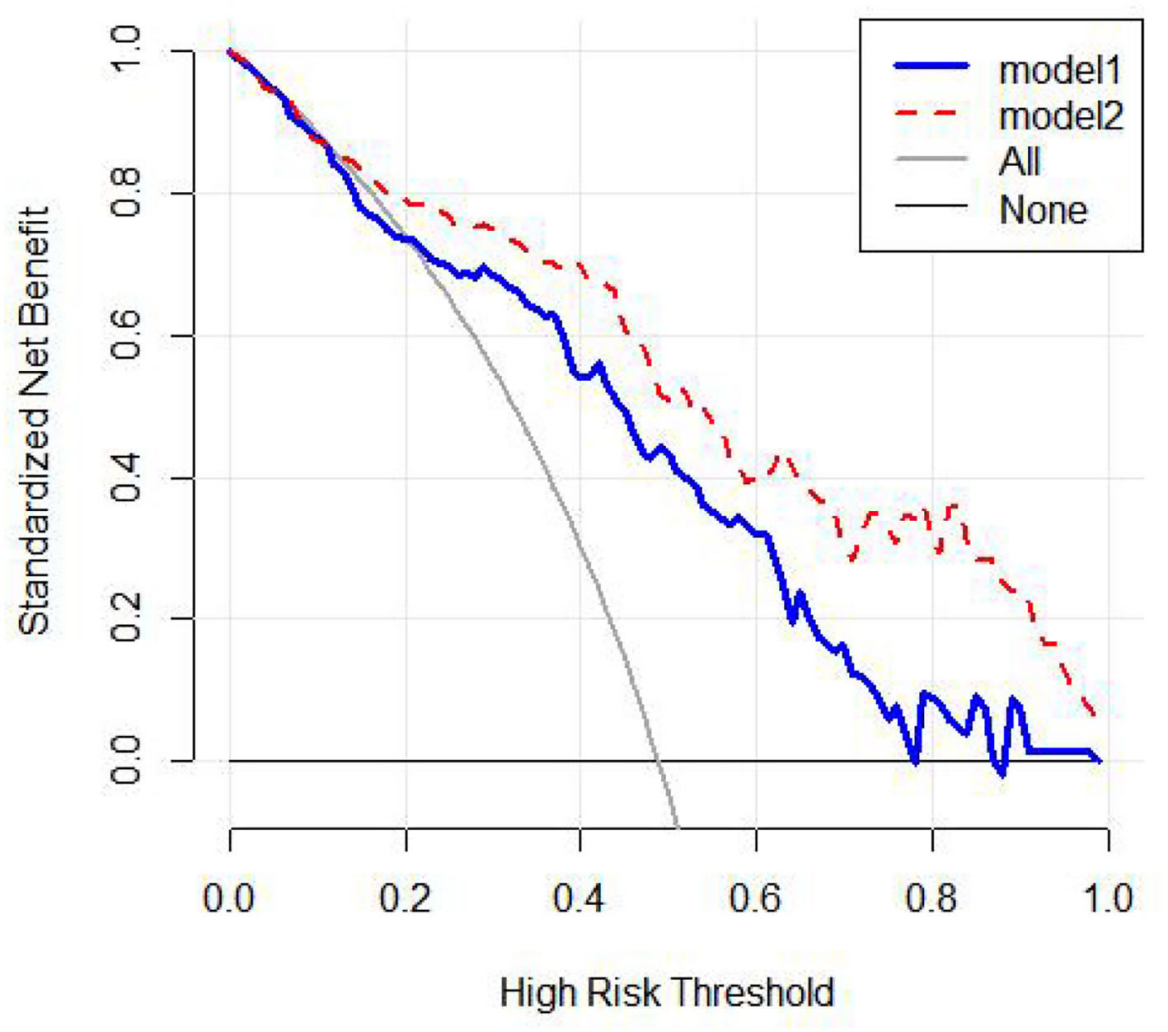
The decision curve analysis for model1 and model2.

**Figure 5:**
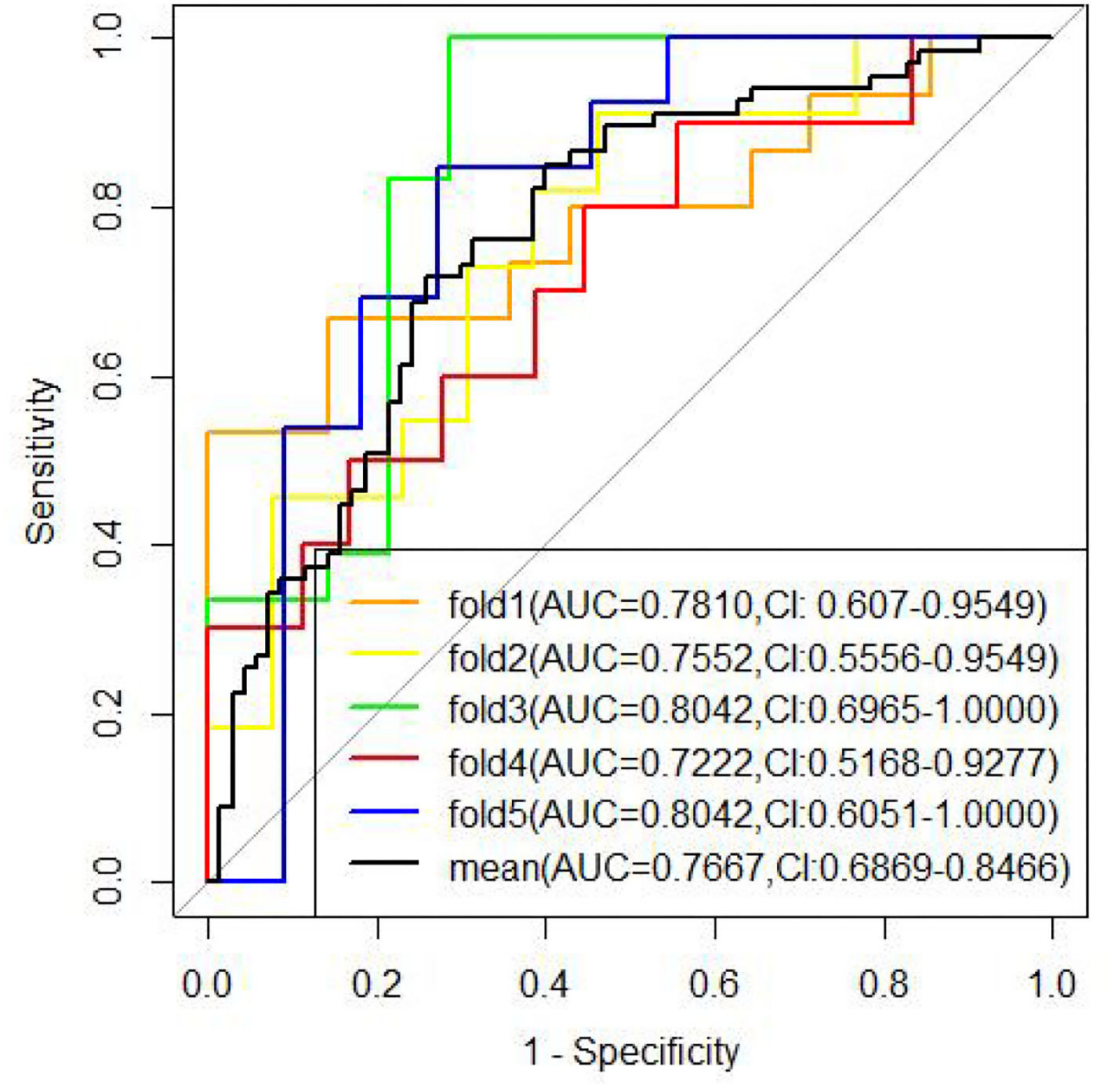
The five cross-validations were used as internal validation for model2.

**Figure 6:**
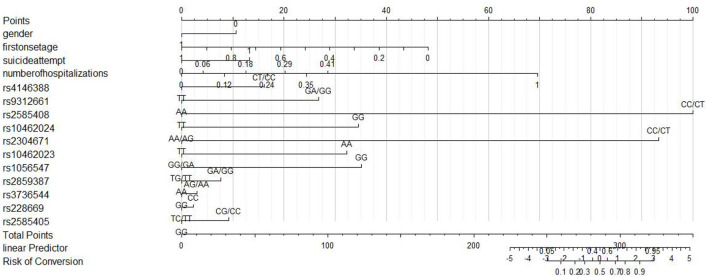
Nomogram for predicting risk of bipolar disorder.

Two models were established: the first model (model1) contained the age of first onset, gender, suicide attempts, and number of hospitalizations; and the second model (model2) included the age of first onset, gender, suicide attempts, number of hospitalizations, rs4146388 in ARNTL, rs1056547, rs9312661, and rs3736544 in CLOCK, rs2585408, rs10462024 and rs2585405 in PER1, rs2304671 and rs10462023 in PER2, rs228669 and rs2859387 in PER3. Their evaluation was performed by AUC value (Figure 2), calibration (Figure 3), NRI, and DCA (Figure 4). The AUC value of model1 was 0.792 (Figure 2A) and that of model2 was 0.779 (Figure 2B). The AUC value of model1 was higher than model2, however, there is no significant difference between the AUC value of model1 and model2 using the delong test (*p*-value = 0.805). The calibration curve of model2 (Figure 3A) was closer to the diagonal dotted line than that of model1 (Figure 3B), suggesting that model2 had a better predictive effect. It could be seen from the DCA curve picture that the curves of model2 are basically above that of model1. It indicated that model2 had better performance than model1. The Akaike information criterion (AIC) of model1 and model2 were 165.95 and 158.17, and the NRI was 0.1851 [0.0207–0.3494] (*p* = 0.027), indicating an improved predictive ability in model2 compared with model1. We chose model2 as the final predictive model based on the above results. Moreover, five cross-validations was used as internal validation for model2 (Figure 5). The mean AUC value was 0.7667 [95% confidence interval (CI): 0.6869–0.8466] and the prediction accuracy was 71%. Finally, a nomogram was drawn (Figure 6) for its predictive ability. According to the value of each risk factor corresponding to the above scale (0–100), we could determine the corresponding score of each risk factor from the nomogram. The total score was the sum of the scores for each factor. From the downward projection of the total score, the corresponding predicted value of the risk of MDD-to-BD conversion could be obtained.

## Discussion

According to these results, the age of onset was the risk factor for MDD-to-BD conversion using logistic regression analysis, RFE, GBDT, and RF. Furthermore, suicide attempts and the number of hospitalizations were also associated with MDD-to-BD conversion. Eleven circadian rhythm gene polymorphisms were associated with MDD-to-BD conversion by feature screening. In this study, the characteristics mentioned above were used to establish two models by ML, and four evaluation methods proved that the model with clinical characteristics and SNPs had the better predictive ability.

The conversion rate of our study was 12.7%, similar to a prospective cohort study which showed the rate of conversion from MDD to BD in 3 years was 12.4% (35). The conversion rates have been reported as between 2.5 and 15.4% in follow-up intervals of 3–9 years (37). The conversion rate of our study was consistent with these studies.

The logistic regression analysis, RFE, and GBDT showed that age at first onset is a risk factor for the disease. We found that the younger the age of onset, the higher the risk of BD, which is consistent with previous studies. Baldessarini et al. found a different rate of switch-risk between adult patients with MDD and adolescent patients with MDD, with the adolescent rate of 5.62%/year (9.33%/1.66 years) and an adult rate of 1.26%/year (3.66%/2.90 years) (38), indicating that conversion is closely associated with the age of onset.

While using GBDT and RF to screen the characteristics, it was found that suicide attempts were also closely related to MDD-to-BD conversion. Previous studies have clustered many risk factors and identified the four most important variables, including suicide attempts (12). It was found that the number of hospitalizations was associated with MDD-to-BD conversion by using RFE, GBDT, and RF to screen the characteristics. Dominika et al. also found that the number of hospitalizations can be the predictive factor (14).

This study provided preliminary evidence that several SNPs of multiple circadian rhythm genes were associated with MDD-to-BD conversion. Some polymorphisms, such as rs2859387 of PER3 and rs4146388 of ARNTL, were related to the occurrence of BD in previous studies. We found rs4146388 in ARNTL was associated with MDD-to-BD conversion. Significant epistasis was found in the ARNTL (rs4146388 and rs7107287) and PER3 (rs2172563), indicating that the sum effects influence the occurrence of BD (39).

Mansour et al. found that the genotype distribution of PER3 rs2859387 was significantly different between BD and healthy control subjects (40). At the same time, our results showed that this SNP is associated with MDD-to-BD conversion. This polymorphism is located in the exon region; it is a synonymous mutation, and how it affects the process of transcription and translation has not yet been clearly determined. However, the sequence change may affect the post-transcriptional processes and regulation, translational efficiency, or protein folding, affecting protein expression and biological function. We have also identified SNPs that have not been found to be associated with affective disorders in previous studies. More research is needed to clarify the correlation between them.

The purpose of the feature selection process is to use an adequate but minimal number of features to achieve optimal prediction results of the model (41). Two methods for feature selection achieved high AUC values. Although the AUC value of RF was lower than others, almost all of the selected features were included in the results of FRE and GBDT, indicating that the results of the three methods have excellent consistency.

In this study, four methods were performed to evaluate the effectiveness of the model. Although the AUC value of the model2 with SNPs showed no difference from that of the model1 without SNPs, three other feature selection methods showed that the predictive ability of the model with both clinical characteristics and SNPs was improved. The AUC value of model2 was lower than model1. It may be due to the fact that the AUC is an insensitive metric when evaluating improvement in predictive performance (42). The AUC value, including genetics, would be substantially lower than a model with clinical characteristics (43). Researchers have used the classification regression tree to establish a predictive model for mood disorders that only included SNPs and had the insufficient predictive ability (39). A risk-stratification model which only included clinical factors was established by the same method, with an AUC of 0.743 (28). The above predictive models only included unilateral factors with insufficient efficacy. At the same time, the clinical and genetic polymorphisms were combined and four methods were used to evaluate the predictive models established in this study. We have found that the predictive ability was better than the previous two models. All showed that combining clinical characteristics and genetic polymorphism is the better choice.

There are some limitations. First of all, the sample size of this study is small; however, we did the power analysis, and this study achieved the requirement of the minimum number of patients. Second, the dropout rate of patients who followed up was relatively high, impacting the sample size. More studies with a larger sample size, more follow-up times, and more extended follow-up periods are needed to be carried out to get a lower dropout rate and validate the predictive model.

## Conclusion

We have found that the age of onset was a risk factor for MDD-to-BD conversion using logistic regression analysis, RFE, GBDT, and RF. Furthermore, suicide attempts and the number of hospitalizations were also associated with MDD-to-BD conversion. We showed that eleven circadian rhythm gene polymorphisms were associated with MDD-to-BD conversion by feature screening. A model with both clinical features and genetic polymorphisms had a better predictive ability. In future research, a follow-up study with a larger sample is needed to verify our predictive model and modify the accuracy of the predictive model.

## Data Availability Statement

The original contributions presented in the study are included in the article/Supplementary Materials, further inquiries can be directed to the corresponding author/s.

## Ethics Statement

The studies involving human participants were reviewed and approved by the Ethical Committee of Zhongda Hospital. Written informed consent to participate in this study was provided by the participants' legal guardian/next of kin.

## Author Contributions

LC: formal analysis, writing—original drafting, writing—reviewing and editing, and validation. YH and BC: formal analysis. TS, ZC, TT, and CG: data curation. SC: data curation and blood collection. WC: formal analysis and reviewing and editing. YY and ZZ: conceptualization and methodology. ZX: conceptualization, methodology, investigation, data curation, project administration, and writing—reviewing and editing. All authors contributed to the article and approved the submitted version.

## Funding

This work was funded by the Natural Science Foundation of Jiangsu Province (No. BK20181272), Jiangsu Provincial Medical Youth Talent (No. QNRC2016825), National Natural Science Foundation of China (Nos. 81301167 and 81971277), and Key Research and Development Program (Social Development) Foundation of Jiangsu Province (No. BE2019714).

## Conflict of Interest

The authors declare that the research was conducted in the absence of any commercial or financial relationships that could be construed as a potential conflict of interest.

## Publisher's Note

All claims expressed in this article are solely those of the authors and do not necessarily represent those of their affiliated organizations, or those of the publisher, the editors and the reviewers. Any product that may be evaluated in this article, or claim that may be made by its manufacturer, is not guaranteed or endorsed by the publisher.

## References

[1] Dell'Osso B D'Addario C Carlotta Palazzo M Benatti B Camuri G Galimberti D. Epigenetic modulation of BDNF gene: differences in DNA methylation between unipolar and bipolar patients. J Affect Disord. (2014) 166:330–3. 10.1016/j.jad.2014.05.020 25012449

[2] Pedrotti Moreira F Cardoso TC Mondin TC Wiener CD de Mattos Souza LD Oses JP. Serum level of nerve growth factor is a potential biomarker of conversion to bipolar disorder in women with major depressive disorder. Psychiatry Clin Neurosci. (2019) 73:590–3. 10.1111/pcn.12896 31170316

[3] Woo YS Shim IH Wang HR Song HR Jun TY Bahk WM. Diagnosis of bipolar spectrum disorder predicts diagnostic conversion from unipolar depression to bipolar disorder: a 5-year retrospective study. J Affect Disord. (2015) 174:83–8. 10.1016/j.jad.2014.11.034 25486276

[4] Menezes IC von Werne Baes C Lacchini R Juruena MF. Genetic biomarkers for differential diagnosis of major depressive disorder and bipolar disorder: a systematic and critical review. Behav Brain Res. (2019) 357–8:29–38. 10.1016/j.bbr.2018.01.008 29331712

[5] Holmskov J Licht R W Andersen K Bjerregaard Stage T Morkeberg Nilsson F Bjerregaard Stage K. Diagnostic conversion to bipolar disorder in unipolar depressed patients participating in trials on antidepressants. Eur Psychiatry. (2017) 40:76–81. 10.1016/j.eurpsy.2016.08.006 27997876

[6] Undurraga J Baldessarini R J Valenti M Pacchiarotti I Tondo L Vazquez G. Bipolar depression: clinical correlates of receiving antidepressants. J Affect Disord. (2012) 139:89–93. 10.1016/j.jad.2012.01.027 22406337

[7] Takaesu Y Inoue Y Ono K Murakoshi A Futenma K Komada Y. Circadian rhythm sleep-wake disorders as predictors for bipolar disorder in patients with remitted mood disorders. J Affect Disord. (2017) 220:57–61. 10.1016/j.jad.2017.05.041 28595099

[8] Jie NF Zhu MH Ma XY Osuch EA Wammes M Theberge J. Discriminating bipolar disorder from major depression based on SVM-FoBa: efficient feature selection with multimodal brain imaging data. IEEE Trans Auton Ment Dev. (2015) 7:320–31. 10.1109/TAMD.2015.2440298 26858825 PMC4743532

[9] Cardoso TA Mondin TC Azevedo LB Toralles LMD de Mattos Souza LD. Is suicide risk a predictor of diagnosis conversion to bipolar disorder? Psychiatry Res. (2018) 268:473–7. 10.1016/j.psychres.2018.08.026 30138860

[10] Ratheesh A Davey C Hetrick S Alvarez-Jimenez M Voutier C Bechdolf A. A systematic review and meta-analysis of prospective transition from major depression to bipolar disorder. Acta Psychiatr Scand. (2017) 135:273–84. 10.1111/acps.12686 28097648

[11] Barbuti M Pacchiarotti I Vieta E Azorin JM Angst J Bowden CL. Antidepressant-induced hypomania/mania in patients with major depression: evidence from the BRIDGE-II-MIX study. J Affect Disord. (2017) 219:187–92. 10.1016/j.jad.2017.05.035 28558366

[12] Dumlu K Orhon Z Ozerdem A Tural U Ulas H Tunca Z. Treatment-induced manic switch in the course of unipolar depression can predict bipolarity: cluster analysis based evidence. J Affect Disord. (2011) 134:91–101. 10.1016/j.jad.2011.06.019 21742381

[13] Uchida M Serra G Zayas L Kenworthy T Hughes B Koster A. Can manic switches be predicted in pediatric major depression? A systematic literature review. J Affect Disord. (2015) 172:300–6. 10.1016/j.jad.2014.09.046 25451429

[14] Dudek D Siwek M Zielinska D Jaeschke R Rybakowski J. Diagnostic conversions from major depressive disorder into bipolar disorder in an outpatient setting: results of a retrospective chart review. J Affect Disord. (2013) 144:112–5. 10.1016/j.jad.2012.06.014 22871536

[15] Park M Kim SA Yee J Shin J Lee KY Joo EJ. Significant role of gene-gene interactions of clock genes in mood disorder. J Affect Disord. (2019) 257:510–7. 10.1016/j.jad.2019.06.056 31323592

[16] Takaesu Y. Circadian rhythm in bipolar disorder: a review of the literature. Psychiatry Clin Neurosci. (2018) 72:673–82. 10.1111/pcn.12688 29869403

[17] Takaesu Y Inoue Y Murakoshi A Komada Y Otsuka A Futenma K. Prevalence of circadian rhythm sleep-wake disorders and associated factors in euthymic patients with bipolar disorder. PLoS ONE. (2016) 11:e0159578. 10.1371/journal.pone.0159578 27442503 PMC4956158

[18] Cho CH Lee HJ Woo HG Choi JH Greenwood TA Kelsoe JR. CDH13 and HCRTR2 may be associated with hypersomnia symptom of bipolar depression: a genome-wide functional enrichment pathway analysis. Psychiatry Investig. (2015) 12:402–7. 10.4306/pi.2015.12.3.402 26207136 PMC4504925

[19] Geoffroy P A. Clock genes and light signaling alterations in bipolar disorder: when the biological clock is off. Biol Psychiatry. (2018) 84:775–7. 10.1016/j.biopsych.2018.09.006 30409265

[20] Valenzuela FJ Vera J Venegas C Munoz S Oyarce S Munoz K. Evidences of polymorphism associated with circadian system and risk of pathologies: a review of the literature. Int J Endocrinol. (2016) 2016:2746909. 10.1155/2016/2746909 27313610 PMC4893437

[21] Bengesser SA Reininghaus EZ Lackner N Birner A Fellendorf FT Platzer M. Is the molecular clock ticking differently in bipolar disorder? Methylation analysis of the clock gene ARNTL. World J Biol Psychiatry. (2018) 19:S21–9. 10.1080/15622975.2016.1231421 27739341

[22] Rocha PM Neves FS Alvarenga NB Hughet RB Barbosa IG Correa H. Association of Per3 gene with bipolar disorder: comment on “Association study of 21 circadian genes with bipolar I disorder, schizoaffective disorder, and schizophrenia”. Bipolar Disord. (2010) 12:875–6. 10.1111/j.1399-5618.2010.00875.x 21176035

[23] Banach E Pawlak J Kapelski P Szczepankiewicz A Rajewska-Rager A Skibinska M. Clock genes polymorphisms in male bipolar patients with comorbid alcohol abuse. J Affect Disord. (2018) 241:142–6. 10.1016/j.jad.2018.07.080 30121446

[24] Antypa N Mandelli L Nearchou F A Vaiopoulos C Stefanis C N Serretti A. The 3111T/C polymorphism interacts with stressful life events to influence patterns of sleep in females. Chronobiol Int. (2012) 29:891–7. 10.3109/07420528.2012.699380 22823872

[25] Dmitrzak-Weglarz M Pawlak J Wilkosc M Miechowicz I Maciukiewicz M Ciarkowska W. Chronotype and sleep quality as a subphenotype in association studies of clock genes in mood disorders. Acta Neurobiol Exp (Wars). (2016) 76:32–42. 10.21307/ane-2017-003 27102916

[26] Belizario G O Junior R G B Salvini R Lafer B Dias R D S. Predominant polarity classification and associated clinical variables in bipolar disorder: a machine learning approach. J Affect Disord. (2019) 245:279–82. 10.1016/j.jad.2018.11.051 30419527

[27] Wollenhaupt-Aguiar B Librenza-Garcia D Bristot G Przybylski L Stertz L Kubiachi Burque R. Differential biomarker signatures in unipolar and bipolar depression: a machine learning approach. Aust N Z J Psychiatry. (2020) 54:393–401. 10.1177/0004867419888027 31789053

[28] Hu YH Chen K Chang IC Shen CC. Critical predictors for the early detection of conversion from unipolar major depressive disorder to bipolar disorder: nationwide population-based retrospective cohort study. JMIR Med Inform. (2020) 8:e14278. 10.2196/14278 32242821 PMC7165312

[29] Han KM De Berardis D Fornaro M Kim YK. Differentiating between bipolar and unipolar depression in functional and structural MRI studies. Prog Neuropsychopharmacol Biol Psychiatry. (2019) 91:20–7. 10.1016/j.pnpbp.2018.03.022 29601896

[30] Shao J Dai Z Zhu R Wang X Tao S Bi K. Early identification of bipolar from unipolar depression before manic episode: evidence from dynamic rfMRI. Bipolar Disord. (2019) 21:774–84. 10.1111/bdi.12819 31407477

[31] Hu S Li A Huang T Lai J Li J Sublette M E. Gut microbiota changes in patients with bipolar depression. Adv Sci (Weinh). (2019) 6:1900752. 10.1002/advs.201900752 31380217 PMC6662053

[32] Poletti S Vai B Mazza M G Zanardi R Lorenzi C Calesella F. A peripheral inflammatory signature discriminates bipolar from unipolar depression: a machine learning approach. Prog Neuropsychopharmacol Biol Psychiatry. (2021) 105:110136. 10.1016/j.pnpbp.2020.110136 33045321

[33] Hamilton M. A rating scale for depression. J Neurol Neurosurg Psychiatry. (1960) 23:56–62. 10.1136/jnnp.23.1.56 14399272 PMC495331

[34] Young RC Biggs JT Ziegler VE Meyer DA. A rating scale for mania: reliability, validity and sensitivity. Br J Psychiatry. (1978) 133:429–35. 10.1192/bjp.133.5.429 728692

[35] Oliveira JP Jansen K Cardoso TA Mondin TC Souza LDM Silva RAD. Predictors of conversion from major depressive disorder to bipolar disorder. Psychiatry Res. (2021) 297:113740. 10.1016/j.psychres.2021.113740 33493732

[36] Abreu T Braganca M. The bipolarity of light and dark: A review on Bipolar Disorder and circadian cycles. J Affect Disord. (2015) 185:219–29. 10.1016/j.jad.2015.07.017 26241867

[37] Biere S Kranz TM Matura S Petrova K Streit F Chiocchetti AG. Risk stratification for bipolar disorder using polygenic risk scores among young high-risk adults. Front Psychiatry. (2020) 11:552532. 10.3389/fpsyt.2020.552532 33192665 PMC7653940

[38] Baldessarini RJ Faedda GL Offidani E Vazquez GH Marangoni C Serra G. Antidepressant-associated mood-switching and transition from unipolar major depression to bipolar disorder: a review. J Affect Disord. (2013) 148:129–35. 10.1016/j.jad.2012.10.033 23219059

[39] Dmitrzak-Weglarz MP Pawlak JM Maciukiewicz M Moczko J Wilkosc M Leszczynska-Rodziewicz A. Clock gene variants differentiate mood disorders. Mol Biol Rep. (2015) 42:277–88. 10.1007/s11033-014-3770-9 25258123

[40] Mansour HA Wood J Logue T Chowdari KV Dayal M Kupfer DJ. Association study of eight circadian genes with bipolar I disorder, schizoaffective disorder and schizophrenia. Genes Brain Behav. (2006) 5:150–7. 10.1111/j.1601-183X.2005.00147.x 16507006

[41] Fan P Guo X Qi X Matharu M Patel R Sakolsky D. Prediction of suicide-related events by analyzing electronic medical records from PTSD patients with bipolar disorder. Brain Sci. (2020) 10:784. 10.3390/brainsci10110784 33121080 PMC7692143

[42] Martens FK Tonk ECM Janssens A. Evaluation of polygenic risk models using multiple performance measures: a critical assessment of discordant results. Genet Med. (2019) 21:391–7. 10.1038/s41436-018-0058-9 29895851 PMC6169739

[43] Chen L Wang YF Liu L Bielowka A Ahmed R Zhang H. Genome-wide assessment of genetic risk for systemic lupus erythematosus and disease severity. Hum Mol Genet. (2020) 29:1745–56. 10.1093/hmg/ddaa030 32077931 PMC7322569

