# Supplementary material for: A Predictive Model of Risk Factors for Conversion From Major Depressive Disorder to Bipolar Disorder Based on Clinical Characteristics and Circadian Rhythm Gene Polymorphisms
Source: Front Psychiatry. 2022 Jul 11;13:843400. doi: 10.3389/fpsyt.2022.843400 (PMC9309512; doi:10.3389/fpsyt.2022.843400)
Supplement: Supplementary file 1 [file Data_Sheet_1.docx]

Supplementary Material

# Supplementary Data

The list of SNPs finally included for statistical analysis

PER3: rs172933, rs228729, rs228642, rs12566042, rs228669, rs79372391, rs228691, rs2859387, rs17031614, rs228701, rs1773135

PER1: rs2585408, rs2585405, rs2289591, rs2253820, rs2735611, rs885747, rs2304911, rs10462024, rs3027178, rs3027172

ARNTL: rs4146388, rs2290037, rs10832030, rs60280155, rs2290034

CLOCK: rs3749472, rs3749473, rs1056547, rs1056545, rs5863, rs6828570, rs6832769, rs10462028, rs6834676, rs1048004, rs1801260, rs3736544, rs11240, rs9312661, rs7684810, rs11133389, rs3817444, rs2272073, rs12505265, rs12505266

PER2: rs56386336, rs934945, rs2304671, rs2304670, rs2304669, rs60242091, rs2304668, rs2304676, rs71426511, rs13033501, rs2304674, rs10462023, rs2304673

# Supplementary Figures and Tables

## Supplementary Tables

Table S1 General characteristics of patients with FU and FFU

|  | FU（N=299） | FFU（N=201） | P value |
| --- | --- | --- | --- |
| gender(male/female) | 88/211 | 59/142 | 0.985 |
| age of onset (mean±SD) | 47.16±15.261 | 44.97±14.390 | 0.110 |
| family history(yes / no) | 42/257 | 19/182 | 0.124 |
| suicide attempt(yes / no) | 156/143 | 103/98 | 0.838 |
| psychotic symptoms(yes / no) | 16/283 | 11/190 | 0.953 |

*FU=patients followed-up successfully, FFU=patients failed to follow-up

Table S2 The general information for all the SNPs

|  | Name | Position | HWpval | %Geno | MAF | Alleles | Rating |
| --- | --- | --- | --- | --- | --- | --- | --- |
| 1 | rs172933 | 7844680 | 0.760 | 97 | 0.388 | G:C |  |
| 2 | rs228729 | 7845695 | 0.951 | 100 | 0.271 | G:C |  |
| 3 | rs3213578 | 7861158 | 1.000 | 94 | 0.021 | C:T | BAD |
| 4 | rs707465 | 7861304 | 0.615 | 86 | 0.477 | C:G | BAD |
| 5 | rs228642 | 7863293 | 0.577 | 100 | 0.190 | G:C |  |
| 6 | rs12566042 | 7864030 | 1.000 | 100 | 0.275 | T:A |  |
| 7 | rs228669 | 7870048 | 0.993 | 100 | 0.287 | G:C |  |
| 8 | rs228671 | 7870418 | 1.000 | 100 | 0.023 | G:C | BAD |
| 9 | rs79372391 | 7870715 | 1.000 | 100 | 0.283 | A:G |  |
| 10 | rs10462018 | 7879627 | 1.000 | 92 | 0.034 | G:C | BAD |
| 11 | rs228691 | 7880469 | 0.951 | 100 | 0.481 | A:T |  |
| 12 | rs2859387 | 7887248 | 0.656 | 100 | 0.194 | A:T |  |
| 13 | rs228697 | 7887579 | 1.000 | 100 | 0.023 | G:T | BAD |
| 14 | rs17031614 | 7887605 | 0.993 | 100 | 0.287 | T:A |  |
| 15 | rs228701 | 7889804 | 0.211 | 100 | 0.167 | T:G |  |
| 16 | rs2640908 | 7889941 | 0.949 | 80 | 0.481 | G:C | BAD |
| 17 | rs12023156 | 7890064 | 0.045 | 100 | 0.178 | A:T | BAD |
| 18 | rs2640909 | 7890117 | 1.000 | 100 | 0.023 | C:G | BAD |
| 19 | rs1773135 | 7890264 | 0.451 | 99 | 0.164 | T:A |  |
| 20 | rs228654 | 7897228 | 1.000 | 100 | 0.023 | T:A | BAD |
| 21 | rs4908699 | 7905274 | 0.271 | 99 | 0.035 | T:A | BAD |
| 22 | rs2585408 | 8043753 | 1.000 | 99 | 0.117 | G:C |  |
| 23 | rs55822222 | 8045061 | 1.000 | 71 | 0.000 | C:C | BAD |
| 24 | rs2585405 | 8046772 | 0.178 | 100 | 0.430 | T:G |  |
| 25 | rs2289591 | 8048010 | 1.000 | 100 | 0.062 | G:A |  |
| 26 | rs2253820 | 8048169 | 1.000 | 100 | 0.388 | C:G |  |
| 27 | rs2735611 | 8048283 | 0.995 | 100 | 0.380 | T:A |  |
| 28 | rs885747 | 8050737 | 0.862 | 100 | 0.120 | T:G |  |
| 29 | rs2304911 | 8050979 | 0.830 | 100 | 0.182 | A:T |  |
| 30 | rs10462024 | 8051639 | 1.000 | 100 | 0.267 | A:T |  |
| 31 | rs3027178 | 8053085 | 0.670 | 100 | 0.260 | C:T |  |
| 32 | rs3027172 | 8055723 | 1.000 | 98 | 0.060 | A:T |  |
| 33 | rs4146388 | 13306605 | 0.629 | 100 | 0.457 | G:C |  |
| 34 | rs2290037 | 13379973 | 0.182 | 100 | 0.167 | C:G |  |
| 35 | rs3816358 | 13391472 | 1.000 | 88 | 0.177 | G:A | BAD |
| 36 | rs10832030 | 13399791 | 0.903 | 100 | 0.403 | T:A |  |
| 37 | rs60280155 | 13400051 | 0.864 | 100 | 0.174 | C:A |  |
| 38 | rs2290034 | 13407184 | 0.539 | 100 | 0.167 | G:C |  |
| 39 | rs3749472 | 56294032 | 0.930 | 94 | 0.079 | T:A |  |
| 40 | rs3749473 | 56294628 | 0.825 | 100 | 0.081 | G:C |  |
| 41 | rs1047354 | 56295583 | 0.038 | 100 | 0.326 | T:A | BAD |
| 42 | rs62303689 | 56295873 | 1.000 | 100 | 0.016 | G:A | BAD |
| 43 | rs1056547 | 56296763 | 0.639 | 91 | 0.368 | T:C |  |
| 44 | rs1056545 | 56296897 | 0.077 | 92 | 0.391 | A:T |  |
| 45 | rs5863 | 56296907 | 0.289 | 98 | 0.370 | T:A |  |
| 46 | rs6828570 | 56297762 | 0.068 | 99 | 0.332 | T:G |  |
| 47 | rs6832769 | 56298194 | 0.074 | 100 | 0.271 | A:T |  |
| 48 | rs10462028 | 56298300 | 1.000 | 100 | 0.058 | T:A |  |
| 49 | rs6834676 | 56298414 | 0.074 | 100 | 0.271 | T:G |  |
| 50 | rs1048004 | 56300209 | 1.000 | 100 | 0.058 | G:A |  |
| 51 | rs3749474 | 56300685 | 0.036 | 95 | 0.373 | C:G | BAD |
| 52 | rs1801260 | 56301369 | 1.000 | 100 | 0.058 | A:T |  |
| 53 | rs3736544 | 56309992 | 0.074 | 100 | 0.271 | T:A |  |
| 54 | rs6811520 | 56315178 | 0.158 | 80 | 0.267 | G:C | BAD |
| 55 | rs11240 | 56319350 | 1.000 | 97 | 0.056 | G:T |  |
| 56 | rs9312661 | 56342326 | 0.051 | 100 | 0.322 | A:T |  |
| 57 | rs7684810 | 56352794 | 0.074 | 100 | 0.271 | C:G |  |
| 58 | rs11133389 | 56352885 | 0.075 | 100 | 0.329 | C:G |  |
| 59 | rs3817444 | 56375981 | 0.091 | 97 | 0.272 | G:A |  |
| 60 | rs2272073 | 56376344 | 0.785 | 96 | 0.363 | C:G |  |
| 61 | rs7691799 | 56411741 | 0.250 | 80 | 0.345 | A:G | BAD |
| 62 | rs12505265 | 56412143 | 1.000 | 99 | 0.051 | T:G |  |
| 63 | rs12505266 | 56412169 | 0.251 | 95 | 0.344 | C:G |  |
| 64 | rs35093813 | 239153765 | 1.000 | 98 | 0.039 | C:A | BAD |
| 65 | rs56386336 | 239153948 | 0.421 | 100 | 0.140 | T:A |  |
| 66 | rs934945 | 239155053 | 1.000 | 100 | 0.256 | G:C |  |
| 67 | rs2304671 | 239165633 | 0.836 | 100 | 0.151 | G:C |  |
| 68 | rs2304670 | 239165636 | 0.545 | 100 | 0.050 | G:C |  |
| 69 | rs2304669 | 239165663 | 0.360 | 100 | 0.136 | C:G |  |
| 70 | rs60242091 | 239165806 | 0.303 | 100 | 0.132 | G:A |  |
| 71 | rs2304668 | 239168448 | 0.545 | 100 | 0.050 | T:A |  |
| 72 | rs2304676 | 239179914 | 1.000 | 100 | 0.306 | T:A |  |
| 73 | rs71426511 | 239180200 | 0.545 | 100 | 0.050 | T:A |  |
| 74 | rs13033501 | 239181860 | 0.545 | 100 | 0.050 | T:A |  |
| 75 | rs2304674 | 239181904 | 0.189 | 100 | 0.322 | A:T |  |
| 76 | rs10462023 | 239184581 | 0.917 | 100 | 0.186 | T:A |  |
| 77 | rs2304673 | 239185922 | 0.524 | 100 | 0.120 | C:T |  |
| 78 | rs2304672 | 239186589 | 0.470 | 100 | 0.047 | T:G | BAD |

BAD: These SNPs have not been included in the statistical analysis.
